# Narrowband diffuse thermal emitter based on surface phonon polaritons

**DOI:** 10.1515/nanoph-2022-0047

**Published:** 2022-03-21

**Authors:** Binze Ma, Yun Huang, Weiyi Zha, Bing Qin, Rui Qin, Pintu Ghosh, Sandeep Kaur, Min Qiu, Qiang Li

**Affiliations:** State Key Laboratory of Modern Optical Instrumentation, College of Optical Science and Engineering, Zhejiang University, Hangzhou 310027, China; Key Laboratory of 3D Micro/Nano Fabrication and Characterization of Zhejiang Province, School of Engineering, Westlake University, 18 Shilongshan Road, Hangzhou 310024, China; Institute of Advanced Technology, Westlake Institute for Advanced Study, 18 Shilongshan Road, Hangzhou 310024, China

**Keywords:** diffuse thermal emitter, narrowband, surface phonon polaritons

## Abstract

Thermal emission engineering with ability to realize spectral and spatial selection has attracted great attention in recent years. Nanophotonic control of thermal radiation has demonstrated narrowband thermal emitter but with high angle-sensitivity and diffuse thermal emitter but with low quality factor (*Q*). Here, we demonstrate a simultaneous narrowband, diffuse thermal emitter consisting of 80 nm (<*λ*/100) thick Ge nanostructures on a silicon carbide (SiC) phononic material. Based on surface phonon polaritons, a spectral coherent emission with a high *Q* factor of 101 is achieved at ∼10.9 μm wavelength in experiment. Furthermore, this phonon-mediated nanostructure provides spatial control with strong diffuse thermal emission with a full angle at half maximum of 70°. Additionally, the emission wavelength and intensity are tuned by replacing Ge with phase change materials (Ge_2_Sb_2_Te_5_ and In_3_SbTe_2_). The designed narrowband diffuse thermal emitter offers new perspectives for the engineering of emission and paves the way for infrared applications, including thermal sources, radiative cooling, infrared sensing, and thermal photovoltaics.

## 1 Introduction

Mid-infrared thermal emitters are crucial for numerous applications, for instance thermal sources [1], radiative cooling [2], [3], [4], [5], [6], infrared sensing [7], [8], [9], thermal photovoltaics [10], and infrared camouflage [11], [12], [13]. Thermal emission is the characteristics of an object with temperature above absolute zero. As temperature increases, the emission intensity increases and the spectral distribution gets blue shifted. The natural thermal emitters such as sun and human body can only provide a broadband diffuse emission (emission at wide angles), which cannot meet the various requirements of thermal emission engineering applications (such as spectral and spatial control). For example, narrowband diffuse thermal emitters are important for improving the sensitivity of gas sensing [1, 7] and enhancing the conversion efficiency of thermal photovoltaics [10]. The conventional strategy to achieve narrowband diffuse emission relies on additional band-pass filters fitted with natural thermal emitters, which makes the whole device bulky and cost-inefficient [14].

**Figure 1:**
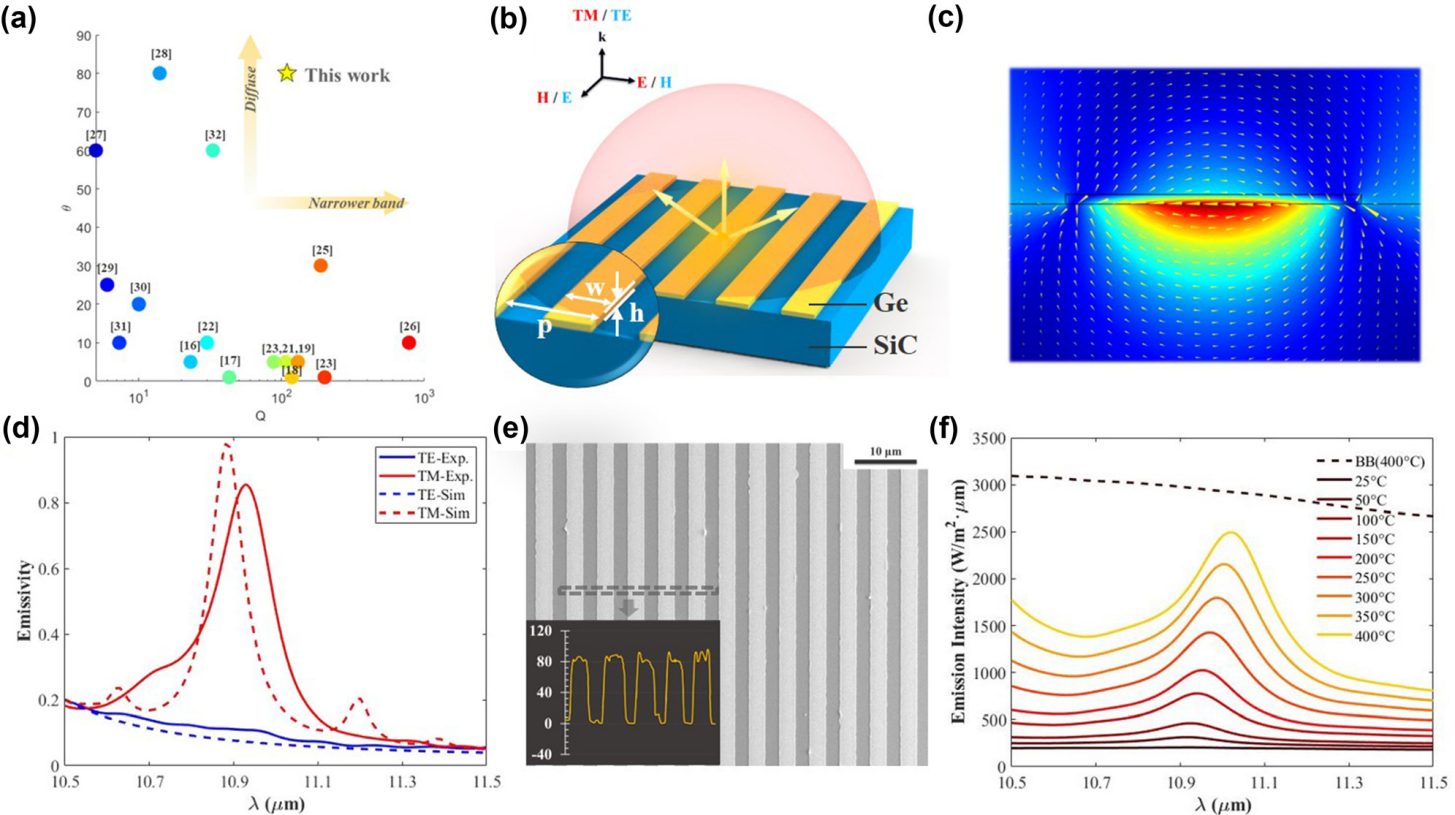
Narrowband diffuse thermal emitter based on phonon-mediated nanostructures. (a) Comparison with reported thermal emitters in terms of spectral and spatial characteristics. (b) Schematic illustration of the designed phonon-mediated nanostructures. (c) Magnetic field distribution of the phonon-mediated nanostructures. The yellow arrows indicate the direction of displacement current. (d) Simulated (dotted curves) and experimental (solid curves) emissivity spectra of the phonon-mediated nanostructure with polarized light. The red curves represent emission under TM polarization and the blue ones under TE polarization. (e) Scanning electron microscopy image of the phonon-mediated nanostructure. (Inset: height-scanning results.) (f) The emission intensity of the phonon-mediated nanostructure under different heating temperatures. The dotted black curve represents the emission intensity of the blackbody under 400 °C.

The development of nanophotonics has facilitated the spectral and spatial control of thermal emission (Figure 1(a) and Table S1 in Supplementary Information) [15], [16], [17], [18], [19], [20], [21], [22], [23], [24], [25], [26], [27], [28], [29], [30], [31], [32], [33], [34], [35], [36]. For spectral control, the desired emission wavelength can be achieved through advanced design of the nanostructures. Various strategies have been adopted to implement narrowband wavelength-selective emitters, such as tungsten bull’s eye structure [18], SiC-based grating [19, 20], photonic crystal [21], and multilayer-film structures based on Tamm plasmon polariton [22], [23], [24], [25], [26]. These emitters can support a strong thermal emission with a high quality factor (*Q* > 100) but limited to a small angular range. For spatial control, metasurface structures have been employed in wide-angle thermal emitters such as metal-insulator-metal metasurface [27], [28], [29], [30], [31] and polar dielectric metasurface [32], [33], [34]. In such structures, thermal emission can be insensitive to direction, with a low *Q* factor (<40). Hence, it is still challenging to realize simultaneous narrowband and diffuse thermal emission.

In this study, we present a synchronized narrowband and diffuse thermal emitter based on surface phonon polaritons (SPhPs). The designed phonon-mediated nanostructures including Ge nanostrips on a SiC substrate exhibit several distinct advantages, comprising (i) narrowband: coherent emission is excited at ∼10.9 μm wavelength with a high *Q* factor of 101; (ii) diffuse thermal emission: phonon-mediated nanostructures can provide a strong emission with a full angle at half maximum of 70°; (iii) ultrathin: the thickness of Ge nanostrips is 80 nm (<*λ*/100); (iv) thermal endurance: the device works favorably till 400 °C; (v) tunability: the resonance wavelength and emission intensity can be tuned by replacing Ge with phase change materials (Ge_2_Sb_2_Te_5_ and In_3_SbTe_2_) as nanostrips. These properties suggest that the phonon-mediated nanostructures can be used as narrowband diffuse thermal emitters for infrared applications, including thermal sources, infrared sensing, and thermal photovoltaics.

## 2 Results

### 2.1 Design and simulation

The narrowband diffuse emitter is designed based on phonon-mediated nanostructures including ultrathin Ge nanostrips on a 6H-SiC polar crystal substrate (Figure 1(b)). The period, width and thickness of nanostrips are 4.9 μm, 2.5 μm and 71 nm, respectively. SiC exhibits metal-like high reflectivity for the wavelength range between 10.32 and 12.61 μm (termed as Reststrahlen band). The long-life-time phonons in Reststrahlen band enable SiC to have lower intrinsic loss (Figure S1(b)) than metal. SiC also presents lower damp rate in comparison with other polar crystals (such as silica and sapphire, shown in Table S2). Combined with lossless Ge nanostrips, the phonon-mediated nanostructures can support a strong magnetic resonance to realize simultaneous narrowband and diffuse thermal emission.

Simulation results show that the distribution of magnetic field and direction of displacement current (Figure 1(c)) manifest a strong magnetic resonance based on SPhPs mode at the interface. It is noted that the magnetic fields of both the resonance and incident light are in the same direction. When the angle varies, the direction of the magnetic field stays the same. Therefore, the phonon-mediated nanostructures can provide a diffuse thermal emission based on the angle-insensitive magnetic resonance. The emission spectrum is examined at normal incidence in the wavelength range of 10.5–11.5 μm (Figure 1(d)). For TM polarization (electric field E perpendicular to the strip), the resonance appears at ∼10.9 μm with a high *Q* factor of 130 (radiative *Q* factor of 320.9 and nonradiative *Q* factor of 218.5, shown in Figure S2). For TE polarization (electric field E along the strip), the resonance cannot be supported and the thermal emitter acts like bulky SiC with high reflectivity and low emissivity.

### 2.2 Thermal emission characteristics

In the experiment, the phonon-mediated nanostructure is fabricated with the size of 4.9 μm, 2.5 μm and 71 nm for the period, width and thickness, respectively (Figure 1(e)). In thermal emission measurement, a narrowband emission with a high *Q* factor of 101 is obtained at ∼10.9 μm wavelength under TM polarization. For TE polarization, the phonon-mediated nanostructure exhibits the characteristics of strong reflection and low emission. The slight discrepancy between the simulated and experimental emissivity can be ascribed to the background emissions in the measurements [37].

The emission intensity of the phonon-mediated nanostructure can be tailored by controlling the device temperature. In the emission measurement (Figure 1(f)), the phonon-mediated nanostructure presents a strong enhancement in the peak emission intensity from 200 to 2500 W/(m^2^·μm) when the working temperature is increased from 25 °C (room temperature) to 400 °C, verifying the thermal endurance of the phonon-mediated nanostructure. In this process, the resonance wavelength maintains at ∼10.9 μm. The slight red shift of emission peak is due to the increase in the refractive index of SiC with increasing temperature [19].

**Figure 2:**
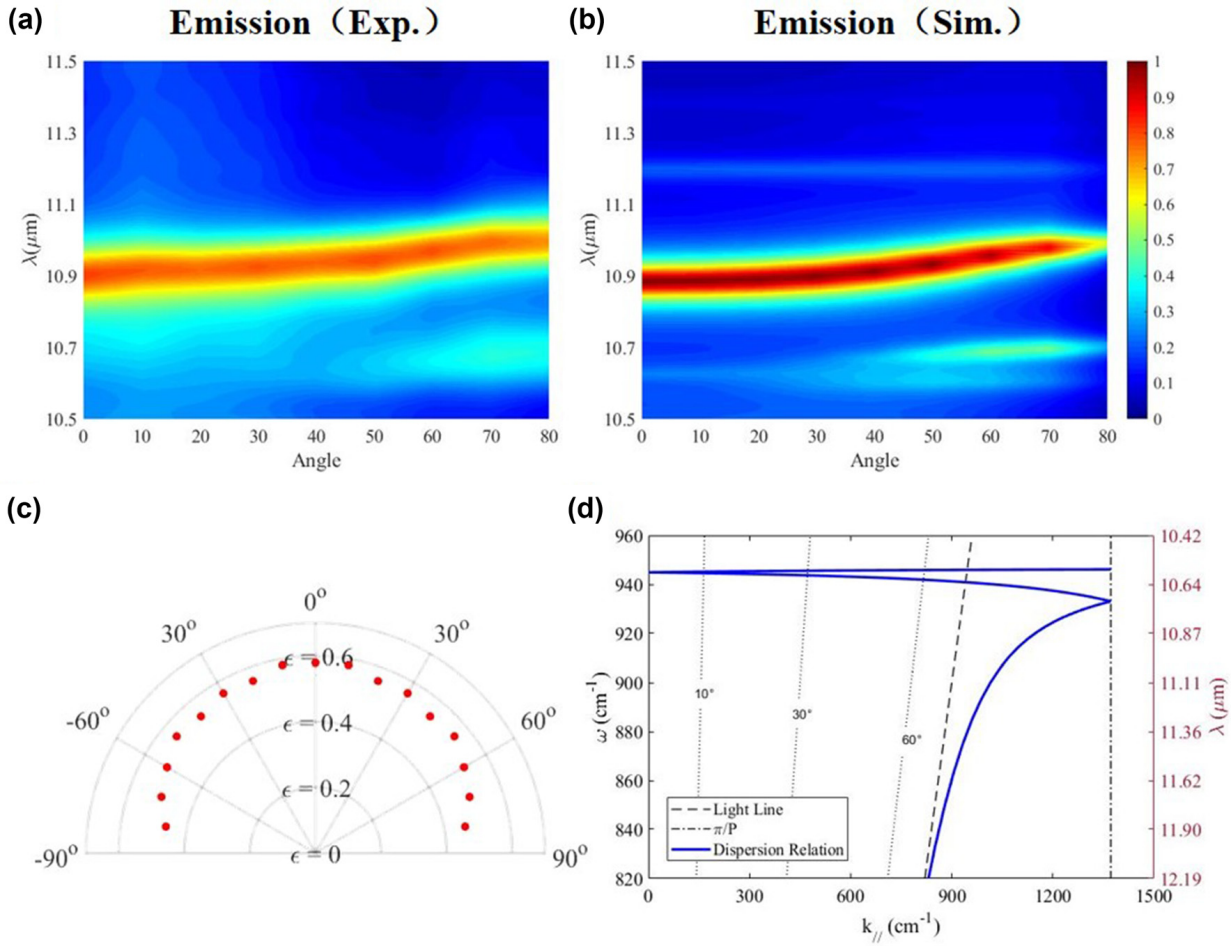
Dependence of thermal emissivity on the angle. (a) and (b) represent the experimental and simulated emission spectrum with different incident angles, respectively. (c) Angular profile of mean emissivity at 10.9 ± 0.1 μm in the experiment. (d) Dispersion relation of the surface phonon polaritons in the designed phonon-mediated nanostructures.

For spatial control, the phonon-mediated nanostructure can support diffuse emission. From the simulated and experimental results (Figure 2(a) and (b)), it is observed that the phonon-mediated nanostructure maintains a high emissivity of ∼0.8 at ∼10.9 μm from 0° to 80°. From the perspective of intensity change at resonance in different directions, a full angle at half maximum is proposed to evaluate the diffuse emission characteristics. The full angle at half maximum of the fabricated phonon-mediated nanostructure is 70° (Figure 2(c)). The diffuse emission property is verified from the calculation of dispersion relation (Figure 2(d) and Supplementary Information Note 3). When the wave vector along the interface (*k*_//_) increases (corresponding to the increasing angles), the resonance wavelength is maintained at ∼10.7 μm (940 cm^−1^). The slight discrepancy of resonant wavelength of dispersion diagram with experimental and simulated results is ascribed to the nonideal approximation of effective permittivity based on effective medium theory (Supplementary Information Note 3).

**Figure 3:**
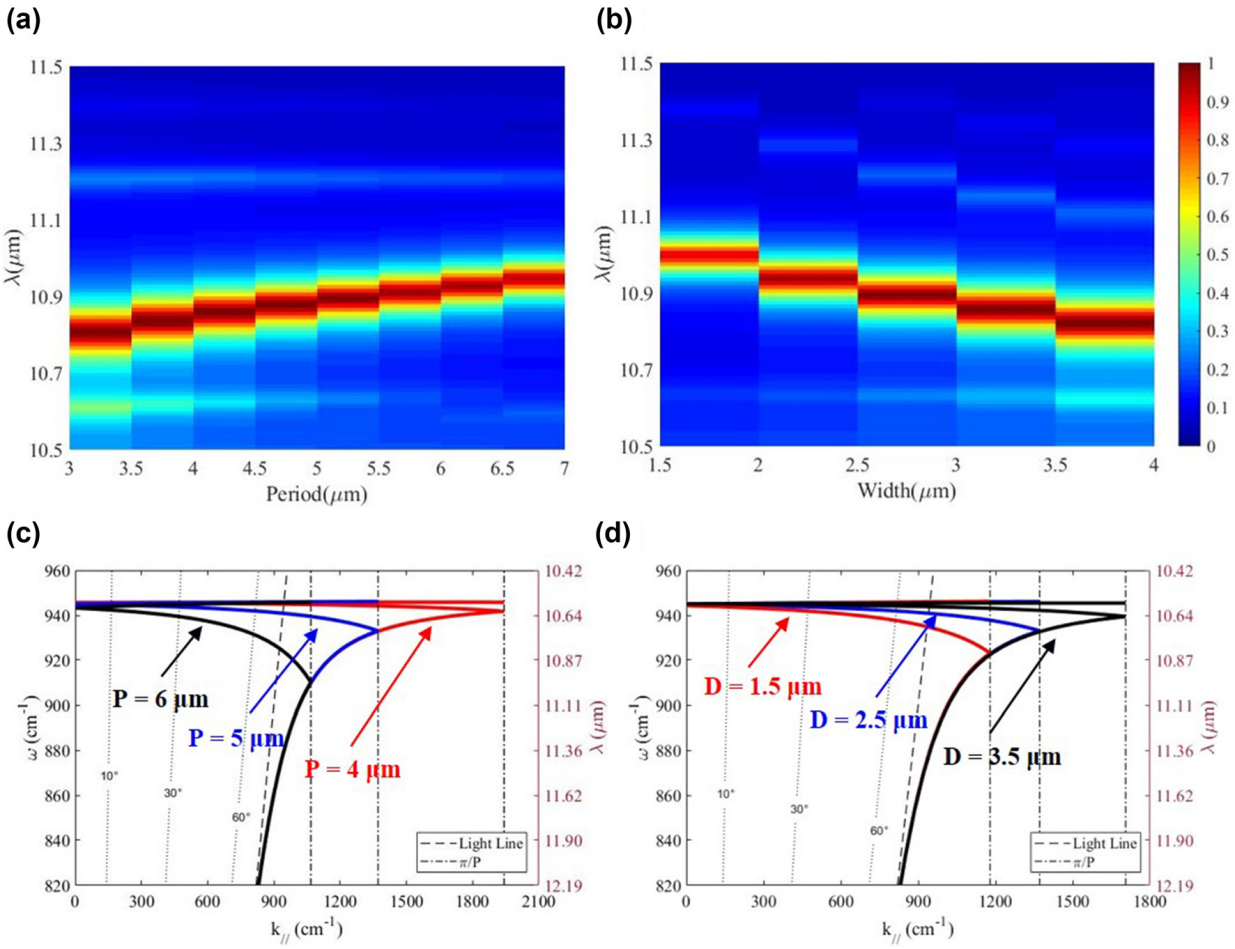
Dependence of thermal emissivity on the geometrical parameters. (a) and (b) Simulated emission spectrum of phonon-mediated nanostructures with changing period and width. In (a), P = 5 μm and H = 80 nm; in (b), D = 2.5 μm and H = 80 nm. (c) and (d) Dispersion relation of surface phonon polaritons with different period and width. (c) Red, blue and black curves represent P = 4, 5 and 6 μm, respectively with D = 2.5 μm. (d) Red, blue and black curves represent D = 1.5, 2.5 and 3.5 μm, respectively with P = 5 μm.

In addition, it can be noted that the resonance wavelength of the phonon-mediated nanostructure is insensitive to the changes in period and width (Figure 3(a) and (b)). As the period increases from 3 to 7 μm, the resonance wavelength changes by 1.2% only (i.e., from 10.85 to 10.98 μm). Also, increment of width from 1.5 to 4 μm leads to a slight shift of the resonance wavelength from 11 to 10.8 μm which is ∼1.9%. During this process, the phonon-mediated nanostructure can maintain a strong emission at ∼10.9 μm, which is also verified by the theoretical calculation of dispersion relation (Figure 3(c) and (d)) and experimental results (Figure S4). With decreasing period and increasing width, the resonance wave vector can be maintained at ∼940 cm^−1^ (∼10.7 μm).

### 2.3 Tunable thermal emission with phase change materials

**Figure 4:**
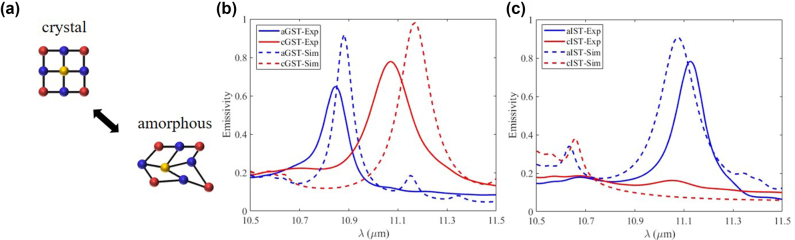
Tunable thermal emission with phase change materials. (a) Schematic illustration of the phase transition. (b) The simulated (dotted curve) and experimental (solid curve) emission spectra of GST-SiC phonon-mediated nanostructure. (c) The simulated (dotted curve) and experimental (solid curve) emission spectra of IST-SiC phonon-mediated nanostructure.

The phonon-mediated nanostructure is observed to provide a tunable thermal emission when Ge is replaced with phase change materials. Ge_2_Sb_2_Te_5_ (GST) and In_3_SbTe_2_ (IST) are two typical phase change materials with large changes in the intrinsic refractive index during the phase transition from amorphous state to crystalline state (Figure 4(a)) [38], [39], [40], [41], [42], [43], [44], [45], [46], [47], [48]. The phase transition of GST and IST is nonvolatile, i.e., the phase state can be maintained when the control stimulus (such as thermal stimulus) is removed [47].

For GST-SiC phonon-mediated nanostructure, a narrowband emission with tunable resonance wavelength is achieved with thermal control. The designed and fabricated GST phonon-mediated nanostructures have sizes of 5.7 μm, 2.5 μm and 76 nm for the period, width and thickness, respectively (Figure S5(a) and (b)). During the phase transition from amorphous state to crystalline state, the resonance wavelength of the GST-SiC phonon-mediated nanostructure goes through a distinct red shift from 10.83 to 11.2 μm (Figure 4(b)). In this process, the *Q* factor of the emission peak remains above 70 and the emissivity stays above 0.6, which is due to the small intrinsic loss of GST in both phase state (Figure S1(e)).

For the IST-SiC phonon-mediated nanostructure emitter, a narrowband diffuse thermal emitter with tunable emissivity is realized with thermal control. The designed and fabricated IST phonon-mediated nanostructures have sizes of 6 μm, 2.3 μm and 161 nm for the period, width and thickness, respectively (Figure S5(c) and (d)). When IST is at amorphous state, the resonance appears at 11.15 μm with an emissivity of ∼0.8 and a *Q* factor of 80 (Figure 4(c)). When IST is transitioned to the crystalline phase, the resonance disappears as the crystalline IST behaves like metal (Figure S1(f)) and the magnetic resonance is no longer supported.

## 3 Conclusion and discussion

In summary, a narrowband diffuse thermal emitter is demonstrated based on phonon-mediated nanostructure. The phonon-mediated nanostructure can support a strong emission with a high *Q* factor of 101 with full angle at half maximum of 70°, which exceed the state-of-the-art thermal emitters in terms of simultaneous narrowband and diffuse properties (see Table S1 in Supplementary Information for detailed comparison). Assisted with phase change materials, including GST and IST, the phonon-mediated nanostructure demonstrates a tunable thermal emission. Firstly, the working wavelength can be extended using other polar crystal materials (such as silica and sapphire). Secondly, *Q* factor of thermal emission can be further improved by using van der Waals materials with lower loss (such as hBN and α-MoO_3_) [49]. Thirdly, the phase transition process can be carried out by light or electricity besides heat, which leads to a faster switching. Furthermore, structural colors can be achieved on narrowband diffuse emitters by depositing silicon thin films without interfering with infrared performance. The designed narrowband diffuse thermal emitter offers new perspectives for the engineering of emission and open the ways for variety of infrared applications, including thermal sources [1, 50], [51], [52], radiative cooling [2], [3], [4], [5], [6], infrared sensing [7], [8], [9], thermal photovoltaics [10], and infrared camouflage [11], [12], [13].

## 4 Materials and methods

### 4.1 Fabrication

The phonon-mediated nanostructures were fabricated on a 6H-SiC substrate by photolithography technique. A layer of photoresist AR-P 5350 was spin-coated on the substrate at 4000 rpm for 60 s and then baked at 150 °C for 5 min. The designed patterns were transferred to the photoresist by photolithography (MA6-BSA). The development was performed in AR 300-26 (1:7) for 18 s followed by rinsing with DI water. The Ge, GST, and IST were deposited by magnetron sputtering (DENTON DISCOVERY-635) at deposition rates of 0.47 nm/s, 0.11 nm/s, and 0.52 nm/s, respectively. Finally, the phonon-mediated nanostructures were obtained by lift-off in acetone.

The as-deposited GST or IST films were initially in the amorphous phase. The phase transitions of GST or IST from amorphous phase to crystalline phase was carried out by heating the sample on a hot plate. For GST, the annealing process was performed at 150 °C for 5 min. And for IST, the annealing process was performed at 300 °C for 5 min. After annealing, the samples with GST or IST films at crystalline phase can be moved out of the hot plate and used for the emissivity measurements.

### 4.2 Emissivity measurements

The emissivity of the phonon-mediated nanostructure was measured by the Fourier transform infrared spectrometer (FTIR, Bruker Vertex 70) equipped with a liquid-nitrogen-cooled mercury cadmium telluride detector (MCT). The emissivity measurements were carried out by heating to induce thermal emission. A black soot was used as the standard blackbody. Both the black soot and the samples were fixed on the heating plate. The emitted power was sent to the FTIR system and detected by MCT detector. The temperatures of both the black soot and the sample were controlled above 100 °C to ensure high emitted power and improve signal-to-noise ratio. Every measurement was repeated 16 times to reduce the noise.

### 4.3 Numerical simulations

Numerical simulations were performed with a radio frequency module (electromagnetic waves, frequency domain) in COMSOL Multiphysics. The parameters of the structure were defined in the text. The emissivities (*E*) of the phonon-mediated nanostructures were derived by calculating the absorptivities (*A*) according to the Kirchhoff’s law which states that the angular spectral absorptivity and emissivity must be equal to each other (*E* = *A*).

## Supplementary Material

Supplementary Material
