# Supplementary material for: Narrowband diffuse thermal emitter based on surface phonon polaritons
Source: Nanophotonics. 2022 Mar 21;11(17):4115–22. doi: 10.1515/nanoph-2022-0047 (PMC11501835; doi:10.1515/nanoph-2022-0047)
Supplement: Supplementary file 1 — Supplementary Material [file j_nanoph-2022-0047_suppl.docx]

Binze Ma^1^, Yun Huang^1^, Weiyi Zha, Bing Qin, Rui Qin, Pintu Ghosh, Sandeep Kaur, Min Qiu* and Qiang Li*

Narrowband Diffuse Thermal Emitter Based on Surface Phonon Polariton

Supplementary Information

1. Comparison between various thermal emitters.

Table S1: Comparison between various thermal emitters.

|  | Structure | Structure | Q | Wavelength | FAHM | Ref | Refm^[[1]](#footnote-1)^ |
| --- | --- | --- | --- | --- | --- | --- | --- |
| **Low Q**  **Directional** | Metasurface | W-SiN-Pt | 6 | 4.25 μm | 25° | [1] | [3] |
|  |  | Au-BCB-Au | 10 | 5.8 μm | 20° | [2] | [4] |
|  |  | Au-Al2O3-Al | 7.3 | 4 μm | 10° | [3] | [5] |
|  | Grating | Nanochannel | 23 | 5.66 μm | 5° | [4] | [16] |
|  |  | TiN 1D-Grating | 43 | 3.06 μm | 1° | [5] | [17] |
| **High Q**  **Directional** | Grating | Tungsten Bull's Eye | 118 | 3.5 μm | 1° | [6] | [7] |
|  |  | SiC 1D-grating | 130 | 11.36 μm | 5° | [7] | [19] |
|  |  | SiC 1D-grating | 200 | 11.6 μm | 1° | [8] | [20] |
|  | Photonic crystal | Quantum well | 107 | 9.1 μm | 5° | [9] | [13] |
|  | TPP^[[2]](#footnote-2)^ Films | Optimized aperiodic multilayered films | 188 | 6 μm | 30° | [10] | [25] |
|  |  | Hybrid metal-OTS^[[3]](#footnote-3)^ | 780 | 4.6 μm | 10° | [11] | [26] |
|  |  | DBR^[[4]](#footnote-4)^-cavity-Au TP | 88 | 4.7 μm | 5° | [12] | [23] |
|  |  | DBR-on-Al TP | 30 | 4 μm | 10° | [13] | [22] |
|  | Metasurface | Mo-Al2O3-Mo | 5 | 6 μm | 60° | [14] | [27] |
| **Low Q**  **Diffuse** |  | Al-Al2O3-Al | <14 | 8.65 μm | 80° | [15] | [28] |
|  |  | SiC metasurface | 33 | 12.2 μm | 60° | [16] | [32] |
| **High Q**  **Diffuse** | Resonant Grating | SiC-Ge Grating | 109 | 10.9 μm | 70° | **This work** | |

1. Material parameters

Materials used in this paper includes Ge, SiC, Ge_2_Sb_2_Te_5_, and In_3_SbTe_2_. In simulation, the optical parameter of Ge is set as n_Ge = 4 from 10.5 μm to 11.5 μm [17]. And for SiC, the optical parameters are obtained from the reflectance spectra measured in FTIR (Fig. S1 (a), (b) and (c)). The optical parameters of Au are obtained from the paper [18] (Fig.S1 (d)). The optical parameters of Ge_2_Sb_2_Te_5_ (GST) and In_3_SbTe_2_ (IST) are obtained from the paper [19] and [20] (Fig.S1 (e) and (f)).

The optical properties *ϵ_SiC_* of silicon carbide in the infrared range can be described by Lorentz model

|  | $\boldsymbol{\epsilon}_{SiC}(\omega)=\boldsymbol{\epsilon}_{\infty}\left( 1+\frac{\omega_{L}^{2}-\omega_{T}^{2}}{\omega_{T}^{2}-\omega^{2}+i\Gamma\omega} \right)$ | (1) |
| --- | --- | --- |

where *ϵ_∞_* represents high-frequency permittivity with *ϵ_∞_*=6.7 cm^-1^, *ω_L_* is LO phonon frequency with *ω_L_ =* 969 cm^-1^, *ω_T_* is TO phonon frequency with *ω_T_ =* 793 cm^-1^ and *Γ* represents the damping rate with *Γ =* 4.76 cm^-1^ [21]. And the quality factor of resonance is inversely proportional to the damping rate [22]. Compared with other polar crystals, SiC has lower damping rate in the mid-infrared range, which results in high Q factor in Ge-SiC phonon-mediated nanostructrures.


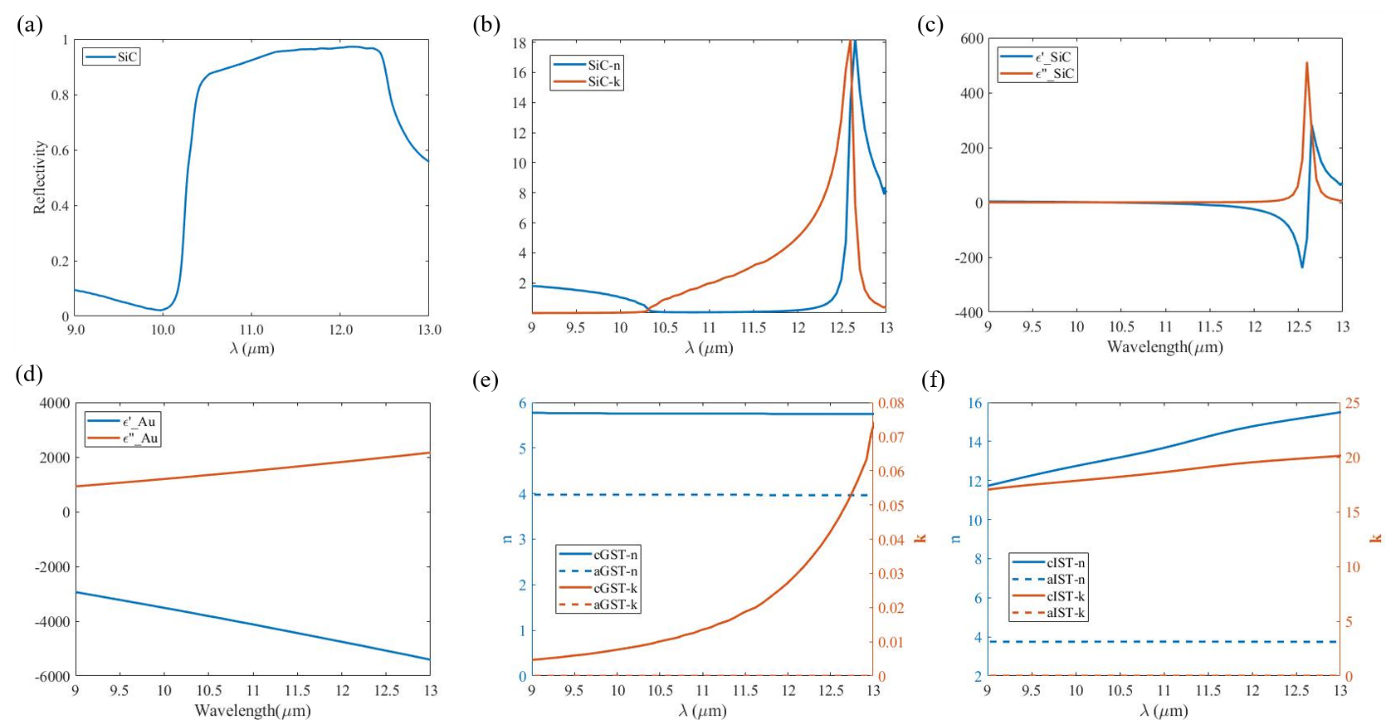


Fig. **S**1: (a) Measured reflectivity of SiC bulk material. (b) The relative refractive index of SiC derived from the reflection spectrum. (c) The relative permittivity of SiC. (d) The relative refractive indices of Al_2_O_3_ and SiO_2_. (e) The relative refractive indices of cGST and aGST. (f) The relative refractive indices of cIST and aIST.

Table S2: Lorentz model parameters of some commonly used polar crystals in the range of 8-14 μm [22].

| polar crystal | ϵ_∞_ | *Γ* (cm^-1^) | *ω_L_* (μm) | *ω_T_*(μm) |
| --- | --- | --- | --- | --- |
| α-Quartz | 2.38 | 182.87 | 8.18 | 8.20 |
|  |  | 7.43 | 7.43 | 9.26 |
|  |  | 7.80 | 12.59 | 12.85 |
| LiF | 1.04 | 22.28 | 10.81 | 32.49 |
| Sapphire | 3.072 | *Γ_L_=15.39* | 11.35 | 17.17 |
| VO_2_ | 10.0 | 42.56 | 13.98 | 14.08 |
| SiC | 6.70 | 4.77 | 10.32 | 12.61 |

1. **Q factor calculation**

The Q factor consists of the radiative part Q_r_ and nonradiative part Q_nr_ via 1/Q=1/Q_r_+1/Q_nr_ [23]. By manually setting the imaginary part of *ϵ_SiC_*, the Q_r_ can be obtained when *ϵ’’* = 0 (Fig.S2).


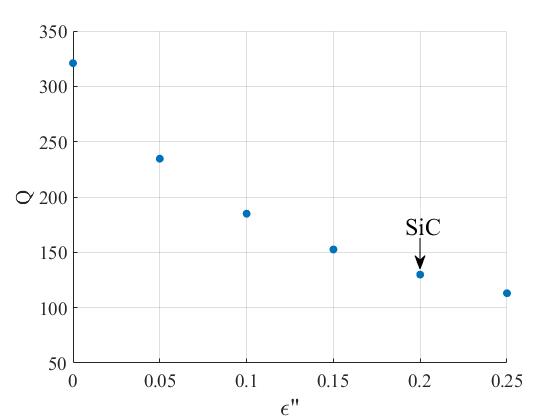


Fig. **S**2: Calculated Q factor of Ge-SiC phonon-mediated nanostructure with different loss parameters.

1. Dispersion relation calculation

In the flat interface between dielectric and SiC, the surface wave follows the dispersion relation as [24]

|  | $tanh\left( u_{d}\epsilon_{d}d \right)=-\frac{1+u_{SiC}/u_{Air}}{\left. u_{d}/u_{Air}+u_{SiC}/u_{d} \right.}$ | (2) |
| --- | --- | --- |

with

|  | $u_{d}=\epsilon_{d}^{-1}\sqrt{{k_{\vert\vert}}^{2}-({\omega^{2}}/{c^{2}})\epsilon_{d}}$ | (3) |
| --- | --- | --- |

where *k_||_* is the parallel wave vector of the surface mode, *ω* is the angular frequency, *c* is light speed in vacuum, *ϵ_d_* is the permittivity of the dielectric. *u_SiC_* and *u_Air_* satisfy similar equations to Equ.(3).

Considering designed structure on SiC, the nanostructures can be equivalent to a thin film with an effective medium permittivity *ϵ_d_* which follows

|  | $\frac{1}{\epsilon_{d}}=\frac{f}{\epsilon_{H}(\omega)}+\frac{1-f}{\epsilon_{L}(\omega)}$ | (4) |
| --- | --- | --- |

where f is the fill factor of nanostructures, *ϵ_H_(ω)* and *ϵ_L_(ω)* presents the permittivity of dielectric and air, respectively. For Ge nanostructures in the paper, Equ.(4) can be solved for *ϵ_d_(ω)*=1.8824, with *ϵ_H_(ω)*=4 and *f* = 0.5. And then with a Bloch mode equation:

|  | $\frac{\omega}{c}\sin\theta=k_{\parallel}+m\frac{2\pi}{\Lambda}$ | (5) |
| --- | --- | --- |

where *θ* is the angle of propagation, *Λ* is the grating period and m is the diffraction order, the dispersion relation can be obtained by solving the Equ.(2) and (3) with complex *ω* and real *k* in a reduced-zone scheme, limited by zero and *π/Λ*. Considering the interfere between dielectric and SiC, *k* includes the intrinsic refractive index with *k* = $\sqrt{\epsilon_{d}}$ *k_||_* and the reduced-zone limited by zero and $\pi/(\Lambda\sqrt{\epsilon_{d}})$ (Fig. S3).


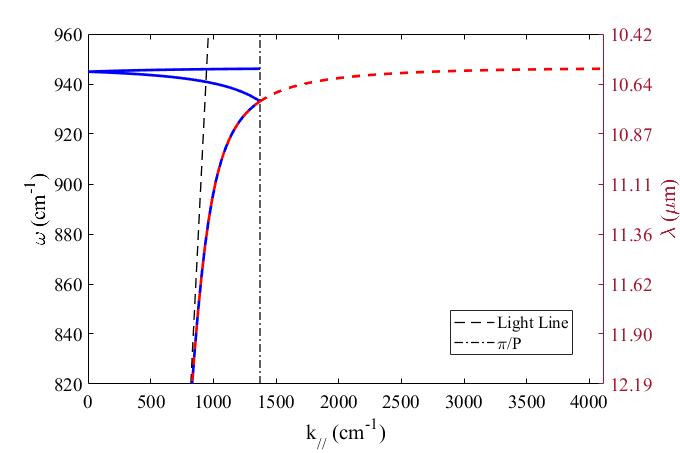


Fig. **S**3: Dispersion relation of surface phonon polariton in the designed phonon-mediated nanostructures.

In dispersion calculation, the Ge nanostructures need to be approximated as a film with an effective permittivity to apply to the dispersion relation. The approximation is based on the effective medium theory, which ignored the high-order terms of effective permittivity [25]. Therefore, the zero-order approximation causes the calculated permittivity to be inconsistent with that of real material, which in turn leads to the slight discrepancy in the resonance wavelength.

1. Experimental results of parameter effect

In experiment, three samples are fabricated with Ge nanostructures of different sizes (Fig.S4 (a), (b) and (c)). The Ge nanostructures in these three samples have the same height of 60 nm. With the sizes change, the resonance wavelength in these three samples are all around 10.8 μm (Fig.S4 (d)).


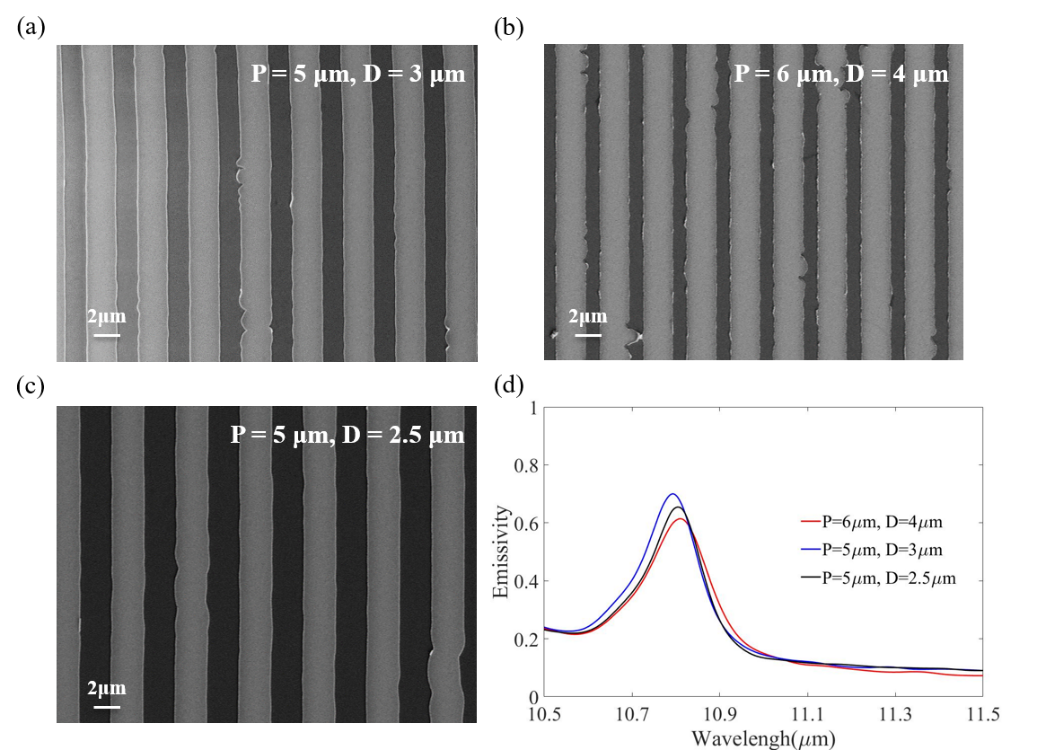


Fig. **S**4: Scanning electron microscopy image of fabricated phonon-mediated nanostructure with (a) P = 5 μm, D = 3 μm; (b) P = 6 μm, D = 4 μm; (c) P = 5 μm, D = 2.5 μm. (d) Emission spectra of the fabricated phonon-mediated nanostructures with TM-polarized light.

1. Fabricated GST-SiC and IST-SiC phonon-mediated nanostructure

The GST-SiC and IST-SiC phonon-mediated nanostructures used in the main text (Fig. 4) are as follows with the scanning electron microscopy image and height-scanning results (Fig. S5). The fabricated GST-SiC phonon-mediated nanostructures have sizes of 5.7 μm, 2.5 μm and 76 nm for the period, width and thickness, respectively. The fabricated IST-SiC phonon-mediated nanostructures have sizes of 6 μm, 2.3 μm and 161 nm for the period, width and thickness, respectively.


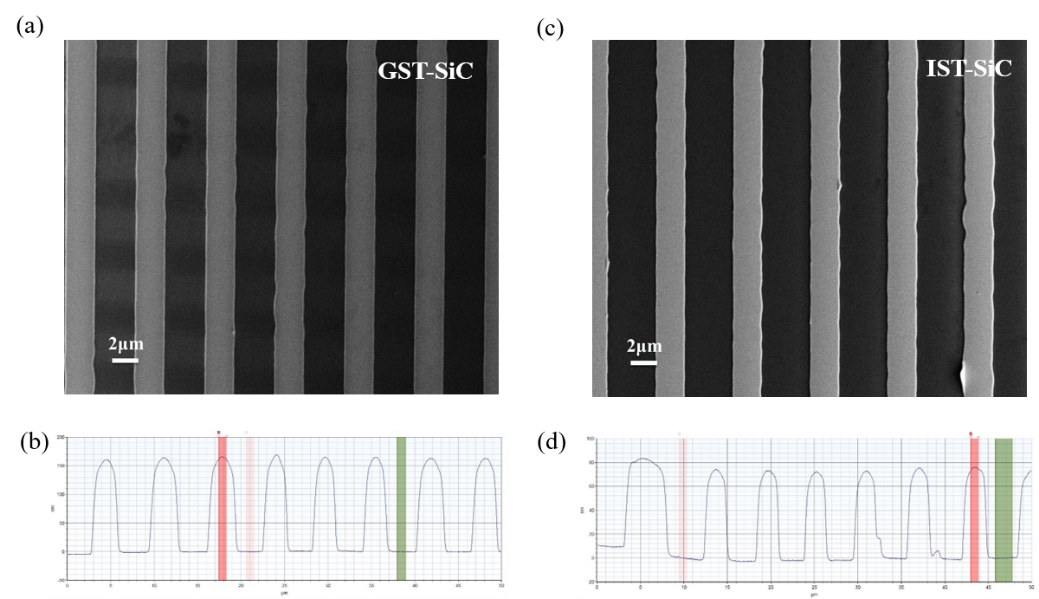


Fig. **S**5: Scanning electron microscopy image and height-scanning results of fabricated GST-SiC (a, b) and IST-SiC (c, d) phonon-mediated nanostructure.

Reference

[1] Wang S, Jiang T, Meng Y, Yang R, Tan G, Long Y. Scalable thermochromic smart windows with passive radiative cooling regulation. *Science* 2021, **374**: 1501–1504.

[2] Heo S-Y, Lee GJ, Kim DH, Kim YJ, Ishii S, Kim MS *et al.* A Janus emitter for passive heat release from enclosures. *Sci Adv* 2020, **6**: 1–9.

[3] Tang K, Dong K, Li J, Gordon MP, Reichertz FG, Kim H *et al.* Temperature-adaptive radiative coating for all-season household thermal regulation. *Science* 2021, **374**: 1504–1509.

[4] Wang Z, Clark JK, Huang L-C, Ho Y-L, Delaunay J-J. Plasmonic nanochannel structure for narrow-band selective thermal emitter. *Appl Phys Lett* 2017; **110**: 251102.

[5] Liu J, Guler U, Lagutchev A, Kildishev A, Malis O, Boltasseva A *et al.* Quasi-coherent thermal emitter based on refractory plasmonic materials. *Opt Mater Express* 2015; **5**: 2721.

[6] Liu N, Tang ML, Hentschel M, Giessen H, Alivisatos AP. Nanoantenna-enhanced gas sensing in a single tailored nanofocus. *Nat Mater* 2011; **10**: 631–636.

[7] Greffet J-J, Carminati R, Joulain K, Mulet J-P, Mainguy S, Chen Y. Coherent emission of light by thermal sources. *Nature* 2002; **416**: 61–64.

[8] Dahan N, Niv A, Biener G, Gorodetski Y, Kleiner V, Hasman E. Extraordinary Coherent Thermal Emission From SiC Due to Coupled Resonant Cavities. *J Heat Transfer* 2008; **130**: 1–5.

[9] Qu Y, Li Q, Cai L, Pan M, Ghosh P, Du K *et al.* Thermal camouflage based on the phase-changing material GST. *Light Sci Appl* 2018; **7**: 26.

[10] Sakurai A, Yada K, Simomura T, Ju S, Kashiwagi M, Okada H *et al.* Ultranarrow-Band Wavelength-Selective Thermal Emission with Aperiodic Multilayered Metamaterials Designed by Bayesian Optimization. *ACS Cent Sci* 2019; **5**: 319–326.

[11] Wang Z, Clark JK, Ho YL, Volz S, Daiguji H, Delaunay JJ *et al.* Ultranarrow and Wavelength-Tunable Thermal Emission in a Hybrid Metal-Optical Tamm State Structure. *ACS Photonics* 2020; **7**: 1569–1576.

[12] Wang Z, Clark JK, Ho Y-L, Vilquin B, Daiguji H, Delaunay J-J. Narrowband Thermal Emission Realized through the Coupling of Cavity and Tamm Plasmon Resonances. *ACS Photonics* 2018; **5**: 2446–2452.

[13] Yang Z-Y, Ishii S, Yokoyama T, Dao TD, Sun M-G, Pankin PS *et al.* Narrowband Wavelength Selective Thermal Emitters by Confined Tamm Plasmon Polaritons. *ACS Photonics* 2017; **4**: 2212–2219.

[14] Yokoyama T, Dao TD, Chen K, Ishii S, Sugavaneshwar RP, Kitajima M *et al.* Spectrally Selective Mid-Infrared Thermal Emission from Molybdenum Plasmonic Metamaterial Operated up to 1000 °C. *Adv Opt Mater* 2016; **4**: 1987–1992.

[15] Dao TD, Chen K, Ishii S, Ohi A, Nabatame T, Kitajima M *et al.* Infrared Perfect Absorbers Fabricated by Colloidal Mask Etching of Al–Al 2 O 3 –Al Trilayers. *ACS Photonics* 2015; **2**: 964–970.

[16] Yang Y, Taylor S, Alshehri H, Wang L. Wavelength-selective and diffuse infrared thermal emission mediated by magnetic polaritons from silicon carbide metasurfaces. *Appl Phys Lett* 2017; **111**: 051904.

[17] Amotchkina T, Trubetskov M, Hahner D, Pervak V. Characterization of e-beam evaporated Ge, YbF 3 , ZnS, and LaF 3 thin films for laser-oriented coatings. *Appl Opt* 2020; **59**: A40.

[18] Olmon RL, Slovick B, Johnson TW, Shelton D, Oh S-H, Boreman GD *et al.* Optical dielectric function of gold. *Phys Rev B* 2012; **86**: 235147.

[19] Du K-K, Li Q, Lyu Y-B, Ding J-C, Lu Y, Cheng Z-Y et al. Control over emissivity of zero-static-power thermal emitters based on phase-changing material GST. Light Sci Appl 2017; **6**: e16194–e16194.

[20] Heßler A, Wahl S, Leuteritz T, Antonopoulos A, Stergianou C, Schön C-F *et al.* In3SbTe2 as a programmable nanophotonics material platform for the infrared. *Nat Commun* 2021; **12**: 924.

[21] Marquier F, Joulain K, Mulet J-P, Carminati R, Greffet J-J, Chen Y. Coherent spontaneous emission of light by thermal sources. *Phys Rev B* 2004; **69**: 155412.

[22] Foteinopoulou S, Devarapu GCR, Subramania GS, Krishna S, Wasserman D. Phonon-polaritonics: enabling powerful capabilities for infrared photonics. *Nanophotonics* 2019; **8**: 2129–2175.

[23] Liu Z, Xu Y, Lin Y, Xiang J, Feng T, Cao Q *et al.* High- Q Quasibound States in the Continuum for Nonlinear Metasurfaces. *Phys Rev Lett* 2019; **123**: 1–6.

[24] Karalis A, Lidorikis E, Ibanescu M, Joannopoulos JD, Soljačić M. Surface-Plasmon-Assisted Guiding of Broadband Slow and Subwavelength Light in Air. *Phys Rev Lett* 2005; **95**: 063901.

[25] D. Marcuse, Theory of Dielectric Optical Waveguides (Academic Press, San Diego, 1991), 2nd ed.

1. Refm: Corresponding reference number in the main text. [↑](#footnote-ref-1)
2. TPP: Tamm plasmon polariton. [↑](#footnote-ref-2)
3. OTS: Optical Tamm state structure. [↑](#footnote-ref-3)
4. DBR: distributed Bragg reflector [↑](#footnote-ref-4)
